# 

**Published:** 1919-10-11

**Authors:** 


### Gifts and Bequests.

At a meeting of the Board of Management of the Manchester Royal Infirmary the following legacies were acknowledged: Mr. Samuel Massey, £133; Mr. John Royle, £300; Mr. David Rope, £200.

The British Red Cross Society, London, have sent surgical dressings and appliances to the value of upwards of £700 to Manchester Royal Infirmary.

Their Majesties the King and Queen have presented bound volumes of *Punch* to the Great Northern Central Hospital, Holloway Road, N.

Mr. Neville Mander, J.P., of the firm of Messrs. Mander Bros., paint manufacturers, Wolverhampton, has bequeathed £500 to the Wolverhampton and Staffs General Hospital.

Ancoats Hospital, Manchester, has received £100 from Mr. F. J. Fitzsimons (in memory of the late Miss Mary Fitzsimons); £100 17s. 6d. from the Manchester City Football Club; and £1,394 4s. 9d., being half the residue of the estate of the late Miss Eliza Mottershead. The amounts received from the Manchester Iron and Steel Merchants' Parade was £214 8s. 2d. The proceeds of the house-to-house collection held on August 30 has realised up to date £200.

Mr. J. O. Lees, of Springhead, Yorks, has bequeathed £300 to Oldham Infirmary and £200 to the Ashton-under-Lyne Infirmary.

Mr. C. W. Stone, of Droitwich, under his will gives £66,000 in trust for his wife during her widowhood, and then for his children, or, on failure of issue, to the Hospital for Sick Children, Great Ormond Street, W.C.

Mr. W. Davidson, of Inverness, has bequeathed £500 to the Bignold Cottage Hospital, £200 to the Edinburgh Royal Infirmary, and £100 each to the Glasgow Royal Infirmary, the Western Infirmary, and Victoria Infirmary.

### Forthcoming Events.

The Society for the Study of Inebriety: Autumn Conference on Tuesday, October 14, 1919, at the Medical Society of London, 11 Chandos Street, Cavendish Square, W. 1.

The Royal Sanitary Institute, 90 Buckingham Palace Road, S.W.—Friday, October 17, at 6.30 p.m., Town Hall, Poole. Discussions on "Influenza," to be opened by A. T. Nankivell, M.D., D.P.H., Medical Officer of Health, Poole, and on "Food Inspection," to be opened by F. St. B. Ramsden, Inspector of Nuisances, Poole, Louis C. Parkes, M.D., D.P.H., Member of Council, in the chair.

### MATRONS' AND SISTERS' APPOINTMENTS.

Grassington Sanatorium, Yorkshire.—Miss Ethel Thomas and Mrs. E. Panton, A.R.R.C., have been appointed sisters. Miss Thomas was trained at the Kent and Canterbury Hospital, has held posts at the General Hospital, Port Said, and has served in Queen Alexandra's Imperial Military Nursing Service Reserve both at home and in France. Mrs. Panton was trained at Hull Royal Infirmary, and has been sister at the Royal Naval Hospital, Hull, and at the Northumberland War Hospital.

Home for Incurables, Liverpool.—Miss Elizabeth Ham has been appointed sister. She was trained at the Mill Road Infirmary, Liverpool, and is a member of the College of Nursing.

City of Westminster Union Infirmary, Hendon.—Miss Edith Worden has been appointed sister. She was trained at the above institution, and has been sister at Endsleigh Palace Hospital, Endsleigh Gardens.

Leeds City Fever Hospital.—Miss Charlotte Lambert has been appointed night sister. She was trained at the Islington Infirmary, and has been sister at the Brook Hospital, Shooter's Hill, sister, 2nd Western Hospital, Manchester, T.F.N.S.

The Infirmary, Beckett Street, Leeds.—Miss Edith M. Sheldon has been appointed third assistant matron. She was trained at the Edmonton Poor-Law Infirmary, where she was afterwards sister, maternity and home sister at West Bromwich Infirmary, and has been on active service at home and abroad.

Children's Hospital, Derby.—Miss A. E. Cresswell has been appointed sister. She was trained at the Royal Infirmary, Sheffield, and has been sister at the Jessop Hospital for Women, sister T.F.N.S. at home and on active service abroad.

Dudley Infirmary.—Miss Edith M. Parker has been appointed ward sister. She was trained at the Union Infirmary, Erdington, and has since been staff nurse under Stone Joint Hospital Board and sister T.F.N.S., 1st North-Western General Hospital, Liverpool.

Charing Cross Hospital.—Miss Routledge has been appointed maternity sister. She was trained at St. Bartholomew's Hospital, where she has since been sister-midwife.

Edge View Tuberculosis Hospital, Kinver, near Stourbridge.—Miss Mabel Mackenzie has been appointed sister. She was trained at St. Marylebone Infirmary and Plaistow Hospital, London, and has since been sister at the Westmorland Sanatorium, Grange-over-Sands, and sister-in-charge at Bourne Castle Sanatorium, Belleroughton, Stourbridge. Miss K. E. Newman has been appointed sister. She was trained at the Stobhill Hospital, Glasgow, and has since been staff nurse and sister under King Edward VII. Welsh National Association.

Infectious Diseases Hospital, Colwyn Bay and Colwyn.—Miss E. Andrews has been appointed nurse-matron. She was trained at Birmingham Poor-Law Infirmary, and has since been sister at Leeds Township Infirmary and nurse-matron at the Isolation Hospital, Cheadle, Stoke-on-Trent, the Peniston District Isolation Hospital, and the Ashford Isolation Hospital, Kent.

East Cliff House, Cliftonville, Margate.—Miss Elvina A. Leeds has been appointed assistant matron. She was trained at St. George's Infirmary, Fulham Road; has since been charge nurse, night superintendent, home sister, and acting assistant matron respectively at the Northern Hospital, Winchmore Hill. Miss M. E. Nuttall has been appointed night sister. She was trained at the Royal Infirmary, Manchester, and the Cancer Hospital, London. She has since been night sister at the Louise Margaret Hospital for Women and Children, Aldershot, and was ward sister at Wrexham Military Hospital. Miss May Boumphrey, Miss Dorothy Richardson, Miss Brigid Rankin, Miss G. M. Gwyn, Miss M. G. Gascoigne, and Miss Lilian Macdonald have been appointed ward sisters. Miss Boumphrey was trained at the David Lewis Northern Hospital, Liverpool, and has since been nursing sister at the King George Hospital, London, and at the Red Cross Hospital, Chipping Norton, and staff nurse at the Alexandra Hospital, Queen Square, Bloomsbury. Miss Richardson was trained at King's College Hospital, London, and the Jones Memorial Hospital for Children, Lancashire; has since been staff nurse and acting sister, T.F.N.S., at 4th London General Hospital, Denmark Hill. Miss Rankin was trained at Victoria Park Hospital for Diseases of the Chest and King's College Hospital, London, and has since been staff nurse and acting sister, T.F.N.S., at the 4th London General Hospital, Denmark Hill. Miss Gwynn was trained at the Royal Sea Bathing Hospital, Margate, and the Essex County Hospital, Colchester, and has since been night sister at the General Hospital, Ramsgate. Miss Gascoine was trained at the West Herts Hospital, Herts, and has since been staff nurse at the West London Hospital, Hammersmith, and at the East London Hospital for Children, Shadwell, and sister at the Surgical Home, Cromwell Road, and in Q.A.I.M.N.S. Miss Macdonald was trained at the Royal South Hants Hospital, Southampton, and Lord Mayor Treloar Cripples Hospital, Alton. She has since been sister at the latter hospital and sister in Q.A.I.M.N.S.

### The College of Nursing
### (Limited by Guarantee).

LONDON CENTRE.

Winter Session, 1919-1920.

The following lectures will be given at 8 p.m. at the rooms of the Medical Society, Chandos Street, Cavendish Square, W. 1:

First Course—By Miss Christie (Historical Tripos, Cambridge), of the London School of Economics.

October 30.—"The Relation of Production to Consumption." November 13.—"Factors in Determining Prices." December 4.—"The Problem of Unemployment."

Second Course—By Miss Bagley, Head of the Polytechnic School of Speech Training.

January 8, 1920.—"Public Speaking: the Voice, its Possibilities, and Power." February 12.—"Public Speaking: the Matter, Ideas, their Sequence and Effect." March 11.—A Debate (subject to be chosen by the audience on January 8) by members of the audience, followed by a criticism by Miss Bagley of the Debate, illustrating points raised in previous lectures.

Members of the London Centre Club admitted on production of card of membership.

Non-members admitted upon payment of 6d. at the door.

### Guy's Hospital Medical School.

The following Entrance Scholarships have been awarded: Senior Science Scholarship for University Students: Arthur Henry Douthwaite, University College, London, £75. Junior Science Scholarships: Thomas Stephenson Blacklidge, Preliminary Science Class, Guy's Hospital Medical School, £120; William Gordon Sears, Preliminary Science Class, Guy's Hospital Medical School, £50. Scholarships in Arts: Donald Fraser Howard, St. Olave's Grammar School, £60; Oswald Fred Farndon, Rhyl County School, £40.

The Treasurer of the Royal Hospital and Home for Incurables, Putney, has received a donation of £1 from the volunteer conductor of a lorry which helped City men and women on their way to business last week during the strike. The donor received more tips than he expected.

